# Membrane-Specific Targeting of Tail-Anchored Proteins SECE1 and SECE2 Within Chloroplasts.

**DOI:** 10.3389/fpls.2019.01401

**Published:** 2019-11-08

**Authors:** Stacy A. Anderson, Rajneesh Singhal, Donna E. Fernandez

**Affiliations:** Department of Botany, University of Wisconsin-Madison, Madison, WI, United States

**Keywords:** chloroplast, inner envelope membrane, thylakoid, organelle biogenesis, SEC translocase, tail-anchored protein, targeting

## Abstract

Membrane proteins that are imported into chloroplasts must be accurately targeted in order to maintain the identity and function of the highly differentiated internal membranes. Relatively little is known about the targeting information or pathways that direct proteins with transmembrane domains to either the inner envelope or thylakoids. In this study, we focused on a structurally simple class of membrane proteins, the tail-anchored proteins, which have stroma-exposed amino-terminal domains and a single transmembrane domain within 30 amino acids of the carboxy-terminus. SECE1 and SECE2 are essential tail-anchored proteins that function as components of the dual SEC translocases in chloroplasts. SECE1 localizes to the thylakoids, while SECE2 localizes to the inner envelope. We have used transient expression in Arabidopsis leaf protoplasts and confocal microscopy in combination with a domain-swapping strategy to identify regions that contain important targeting determinants. We show that membrane-specific targeting depends on features of the transmembrane domains and the short C-terminal tails. We probed the contributions of these regions to targeting processes further through site-directed mutagenesis. We show that thylakoid targeting still occurs when changes are made to the tail of SECE1, but changing residues in the tail of SECE2 abolishes inner envelope targeting. Finally, we discuss possible parallels between sorting of tail-anchored proteins in the stroma and in the cytosol.

## Introduction

For biogenesis and maintenance of a functional chloroplast, proteins must be localized in the correct compartments. Most chloroplast proteins are encoded by nuclear genes. Therefore, targeting is a multi-step process, involving delivery of newly synthesized precursor proteins to the import apparatus in the envelope membranes, translocation through the outer and inner envelopes, and subsequent sub-organellar targeting of the imported proteins (for recent reviews, see Lee et al., 2017; Richardson et al., 2017). Imported proteins have five possible destinations: soluble proteins may reside in the stroma, in the thylakoid lumen, or in the intermembrane space, while membrane proteins may function either in the inner envelope or the thylakoid membranes.

Membrane proteins that are imported present a special challenge: although most membrane proteins are targeted co-translationally in the cyanobacterial ancestors of chloroplasts, they must be targeted in a post-translational fashion within the organelle. Many of these proteins have highly hydrophobic domains and must be shielded from the aqueous environment to prevent aggregation while they are being targeted and integrated. Despite their importance for photosynthesis, biosynthesis, and transport, we know relatively little about the targeting information and systems that direct them to particular membranes. For example, we do not yet have a reliable strategy for directing novel or engineered membrane proteins to the inner envelope (Rolland et al., 2017).

To further our understanding of membrane-specific targeting in chloroplasts, we have studied targeting of the membrane components of the two SEC translocases in chloroplasts. We previously reported on a study of the SCY components (homologs of bacterial SecY proteins) with 10 transmembrane domains (TMDs) (Singhal and Fernandez, 2017). We identified sequences within the N-terminal leader and characteristics of the TMDs as important determinants for membrane-specific targeting, and performed experiments that implicated the chloroplast Signal Recognition Particle (SRP) pathway in the targeting of thylakoid-localized SCY1. In the present study, we have focused on the SECE components (homologs of bacterial SecE proteins). Previous studies have shown that SECE1 is confined to the thylakoids (Schuenemann et al., 1999), while SECE2 is primarily associated with the inner envelope (Li et al., 2015). Both proteins have a single TMD followed by a tail of 30 amino acids or fewer, and belong to a special class of proteins known as tail-anchored (TA) proteins. Because they share a similar structure, we were able to swap different domains without perturbing the overall topology of the proteins. To identify regions important for targeting, we expressed and localized fluorescently tagged chimeric proteins in transfected protoplasts. We learned that targeting determinants are located in the TMD and tail regions, rather than the stroma-exposed N-terminal regions. Physicochemical properties of the TMDs and tails appear to dictate which pathway the TA protein will enter and whether it will insert into the target membrane or remain in a stromal pool. The position and nature of these determinants are fundamentally different than anything previously described in studies of membrane targeting inside chloroplasts. They may, however, include some characteristics that mirror those of cytosolically targeted TA proteins. We suggest that targeting pathways novel to the chloroplast may be involved.

## Materials and Methods

### Sequence Analysis and Definition of SECE Protein Regions

Putative SECE1 and SECE2 protein sequences from multiple plant species were acquired by homology searches using the blastp algorithm of BLAST (https://blast.ncbi.nlm.nih.gov/Blast.cgi) (Altschul et al., 1990) and the predicted protein sequence of *Arabidopsis thaliana* SECE1 (At4g14870) or SECE2 (At4g38490). Protein sequences were acquired from TAIR (https://www.arabidopsis.org/index.jsp). Sequences used for alignments were chosen based on the availability of complete protein sequences for both SECE1 and SECE2 in a given species and to reflect species diversity. Sequences were aligned using the M-Coffee algorithm (Wallace et al., 2006) (http://tcoffee.crg.cat/apps/tcoffee/index.html). The resulting alignment files were submitted to BoxShade (https://embnet.vital-it.ch/software/BOX_form.html) to create the alignment figures.

Different regions of SECE1 and SECE2 were defined based on a combination of topological features and protein sequence alignments. The signature, linker and TMD, and tail regions of SECE2 were previously defined in Li et al. (2015) and comparable regions of SECE1 regions were identified using sequence alignments. The N region was defined as everything N-terminal to the signature region. The N region was further subdivided such that the N1 region contained the predicted transit peptide, the GFP insertion site, and 5–9 amino acids C-terminal to that site. The N2 region included amino acid sequences that showed a moderate degree of conservation in alignments and were predicted to have a helical secondary structure, when analyzed using the JPred4 algorithm (http://www.compbio.dundee.ac.uk/jpred/) (Drozdetskiy et al., 2015). The TMDs were identified using consensus transmembrane domain prediction programs (ConPred_v2 and AramTmCon) available through the Aramemnon database (http://aramemnon.uni-koeln.de/) (Schwacke et al., 2003) or the topology prediction in UniProt (https://www.uniprot.org/) (TheUniprot Consortium, 2019).

The helical propensity, hydrophobicity scores of the TMDs, and hydrophobicity plots were calculated using various web-based tools. Helical propensity was calculated using the Agadir algorithm (http://agadir.crg.es/) (Muñoz and Serrano, 1997) with conditions pH 7.5, 298 K, and ionic strength 0.15 M. The grand average of hydropathy (GRAVY) scores were calculated using the GRAVY calculator (http://www.gravy-calculator.de/) (Kyte and Doolittle, 1982). Hydrophobicity plots for the C-terminal regions (starting with the conserved E-W-P motif) of SECE1 and SECE2 were created using the ProtScale tool in ExPASy (https://web.expasy.org/protscale/) (Gasteiger et al., 2005). The “Hydrophob./Kyte & Doolittle” amino acid scale, a window size of 9, relative weight of 100%, linear weight variation model, and “no normalization of the scale” were used.

### Generation of GFP-Fusion Constructs

Generation of the GFP-SECE2 construct was described previously (Li et al., 2015). For amplification of SECE1 sequence, cDNA prepared from Arabidopsis (Wassilewskija ecotype) seedling RNA or, because SECE1 has no introns, a previously described Columbia ecotype SECE1 genomic clone (Skalitzky et al., 2011) were used as templates. The GFP-SECE1 construct was assembled by amplifying a fragment encoding the putative transit peptide plus the first 33 amino acids of the mature protein, a second fragment encoding GFP, and a third fragment encoding the rest of the protein, and then connecting the fragments using overlap-extension PCR. The final PCR product was then introduced into the vector pML94 (Bionda et al., 2010) between the Kpn1 and BamHI restriction sites by sequence- and ligation-independent cloning (Jeong et al., 2012). pML94 includes sequence encoding the promoter for the 35S gene of cauliflower mosaic virus, which should confer high level constitutive expression *in planta*. All other SECE1–SECE2 chimeric constructs were generated from the GFP-SECE1 and GFP-SECE2 clones using primers that spanned the SECE1–SECE2 junctions. Individual fragments were joined by overlap-extension PCR and the complete coding sequence was introduced into pML94 by sequence- and ligation-independent cloning. All plasmids used for imaging were verified by sequencing.

### Protoplast Isolation and Transfection

For protoplast preparation, *Arabidopsis thaliana* (Columbia ecotype) plants were grown using a 12 h light/12 h dark cycle at 22°C as described in Singhal and Fernandez (2017). Leaves were removed from plants that were 4–5 weeks old and still in the vegetative phase. Protoplasts were isolated using a tape sandwich method as described in Wu et al. (2009) except that more leaves (∼25) were used so that transfections could be performed on a higher number of cells. PEG-mediated transfections of 100,000–500,000 cells were performed as described in Yoo et al. (2007) except that buffer W1 was eliminated and replaced by an equal volume of W5 buffer. Transfections were performed using 10 µg DNA for all plasmids encoding SECE constructs or 6 µg of the TIC20-mCherry plasmid, which was previously described in Singhal and Fernandez (2017). Protoplasts were incubated in the dark for 12–16 h.

### Microscopy and Image Analysis

Transfected protoplasts that had been incubated overnight were transferred to µ-Slide Angiogenesis slides (Ibidi, https://ibidi.com/) and imaged using Zeiss LSM 710 and LSM 780 Confocal microscopes (Zeiss, https://www.zeiss.com/microscopy/us). A 488-nm laser was used for GFP excitation and a 561-nm laser for chlorophyll excitation and mCherry excitation. GFP emission was recorded at 499–526 nm, chlorophyll autofluorescence was recorded at 655–735 nm, and mCherry emission was recorded at 589–636 nm. For each construct, at least three to five transfected protoplasts were imaged in each of three independent experiments.

Co-localization analysis and calculation of Pearson’s correlation coefficients were performed on 16 protoplasts using the Coloc2 plugin of the ImageJ software. Although similar distributions of fluorescent proteins were observed regardless of the position of the chloroplast within the protoplast or its orientation, for fluorescence intensity plots, we chose chloroplasts angled such that distinct thylakoid and stromal regions were visible. Appropriate linear regions were selected and the pixel intensity values were recorded from the GFP and chlorophyll or mCherry channels. Intensity measurements were performed in ImageJ (Version 2.0.0-rc-68/1.52g, NIH, https://imagej.nih.gov). A minimum of three fluorescence intensity plots, one each from three different protoplasts, were generated and compared for each construct. The protoplasts and scans shown are representative images.

## Results

### SECE1 and SECE2 Localize to Distinct Compartments When Transiently Expressed in Protoplasts

**Figure 1:**
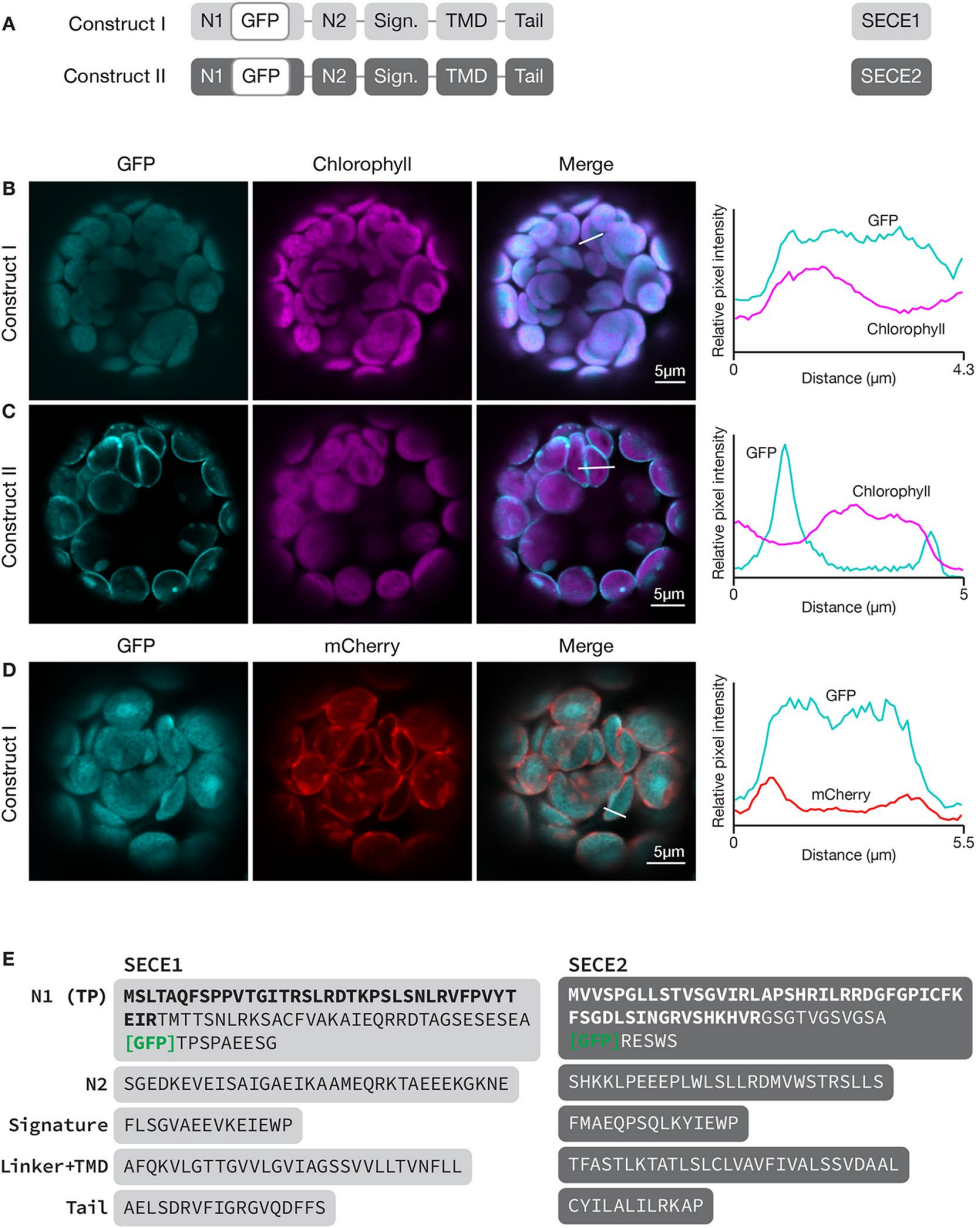
Localization of GFP-SECE1 and GFP-SECE2 in transfected protoplasts and designation of protein domains in SECE1 and SECE2. **(A)** Diagrams depicting constructs I and II. SECE1 sequences are indicated with light gray and SECE2 sequences with dark gray; N1 and N2: N-terminal regions; GFP: green fluorescent protein; Sign.: signature domain; TMD: linker plus transmembrane domain; Tail: C-terminal tail. **(B**–**D)** Leaf protoplasts from 4–5 week old wildtype (Columbia ecotype) plants were transfected with **(B)** construct I, **(C)** construct II, or **(D)** construct I plus a TIC20-mCherry construct. The images show GFP fluorescence (cyan), chlorophyll fluorescence (magenta) or TIC20-mCherry fluorescence (red), merged images, and relative pixel intensity diagrams that correspond with the white lines on the merged images. **(E)** Amino acid sequences that correspond to the individual domains of SECE1 and SECE2. Predicted transit peptides (TP) are shown in bold text and the GFP insertion site is indicated. TMD, transmembrane domain.

To localize SECE1 and SECE2 in chloroplasts, we imaged fluorescent fusion proteins in transfected Arabidopsis leaf protoplasts. Localizations to the stroma, thylakoids, or envelope membranes can be easily distinguished using this assay system (**Figure S1**). To localize SECE1 and SECE2, sequence encoding GFP was added downstream of the predicted transit peptide cleavage site, such that GFP would be in the N-terminal domain of the predicted mature protein and exposed in the stroma. SECE1 has been previously shown by fractionation to be confined to the thylakoids (Schuenemann et al., 1999). For GFP-SECE1 (construct I, **Figure 1A**), the GFP fluorescence occupied most of the interior of the chloroplast, showing strong co-localization (Pearson’s R value = 0.84 ± 0.05) with chlorophyll autofluorescence (**Figure 1B**). However, we also saw some association with interior regions without chlorophyll, which suggests that transfer of the fusion protein from a stromal pool to the thylakoids is less than 100% efficient under our incubation conditions. The relative amounts of GFP-SECE1 in membrane and soluble fractions prepared from chloroplasts isolated from transfected protoplasts are consistent with this conclusion (**Figure S2C**). To demonstrate that GFP-SECE1 is not associated with the inner envelope, constructs encoding GFP-SECE1 and TIC20-mCherry, an inner envelope marker, were co-transfected. The profile of TIC20-mCherry shows two peaks in linear scans, indicating envelope localization, and was spatially separate from the GFP-SECE1 signal (**Figure 1D**). For GFP-SECE2 (construct II), GFP fluorescence was confined to the periphery of the chloroplasts, producing a profile of two distinct peaks in linear scans of individual chloroplasts (**Figure 1C**). The chlorophyll autofluorescence signal showed a very different profile, occupying a more central location to the inside of the GFP-SECE2 peaks. The peripheral localization of GFP-SECE2 is consistent with localization in the inner envelope, which was previously established by demonstrating interactions between SECE2 and the inner envelope integral membrane protein SCY2, as well as co-localization of GFP-SECE2 and TIC20-mCherry in transfected protoplasts (Li et al., 2015). We found that GFP-SECE2 fusion proteins were associated exclusively with the membrane fraction in chloroplasts isolated from transfected protoplasts (**Figure S2D**). We conclude that the distribution of GFP fusion proteins accurately reflects the localization of the chloroplast SECE proteins in different chloroplast compartments.

### SECE1 and SECE2 Have Conserved Structures But Different Sequences

**Figure 2:**
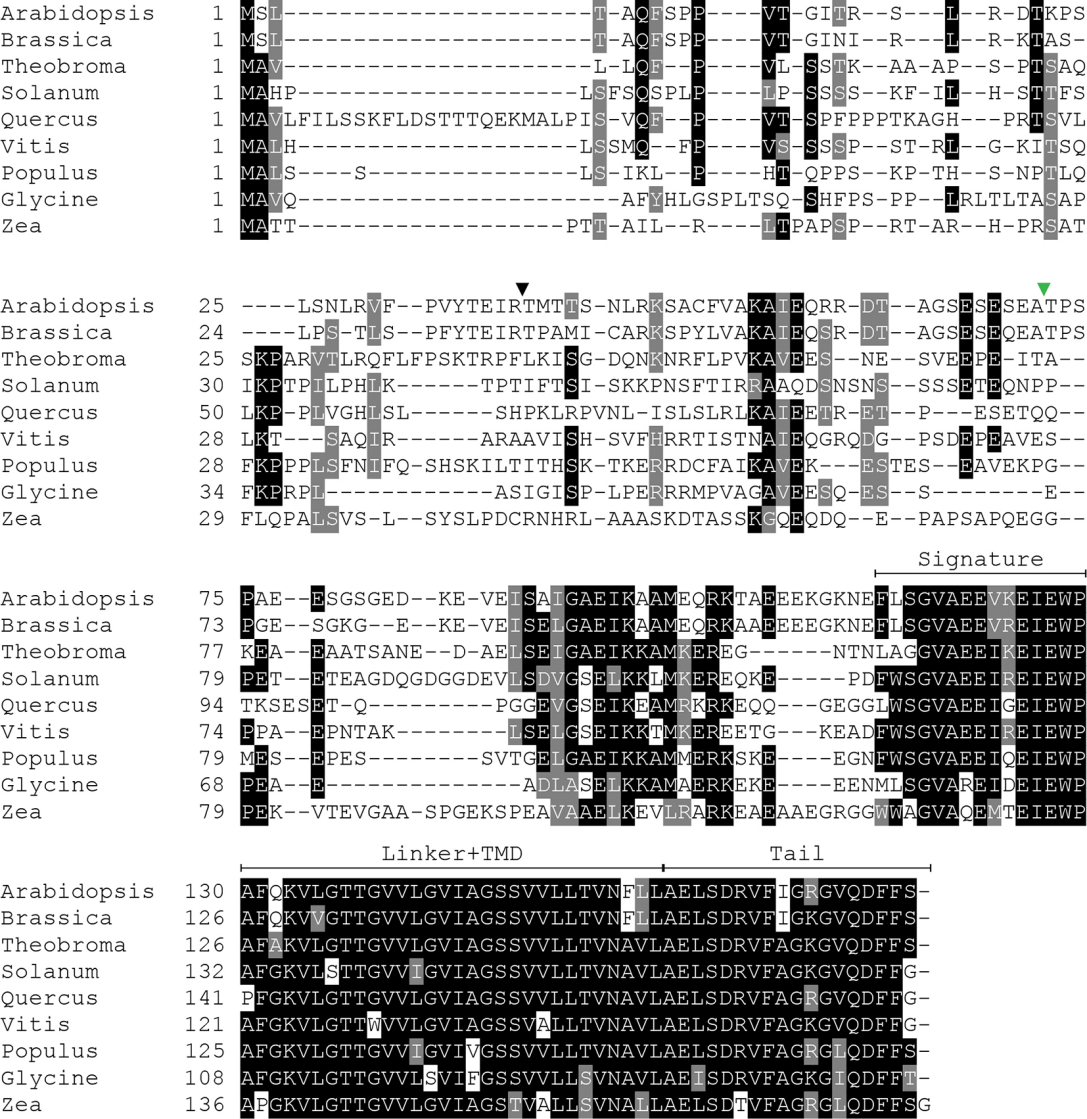
Multiple sequence alignment of SECE1 from a variety of plant species. The predicted transit peptide cleavage site in Arabidopsis is indicated with a black triangle. The GFP insertion site is indicated by a green triangle. Signature, signature region; TMD, transmembrane domain; Tail, C-terminal tail.

**Figure 3:**
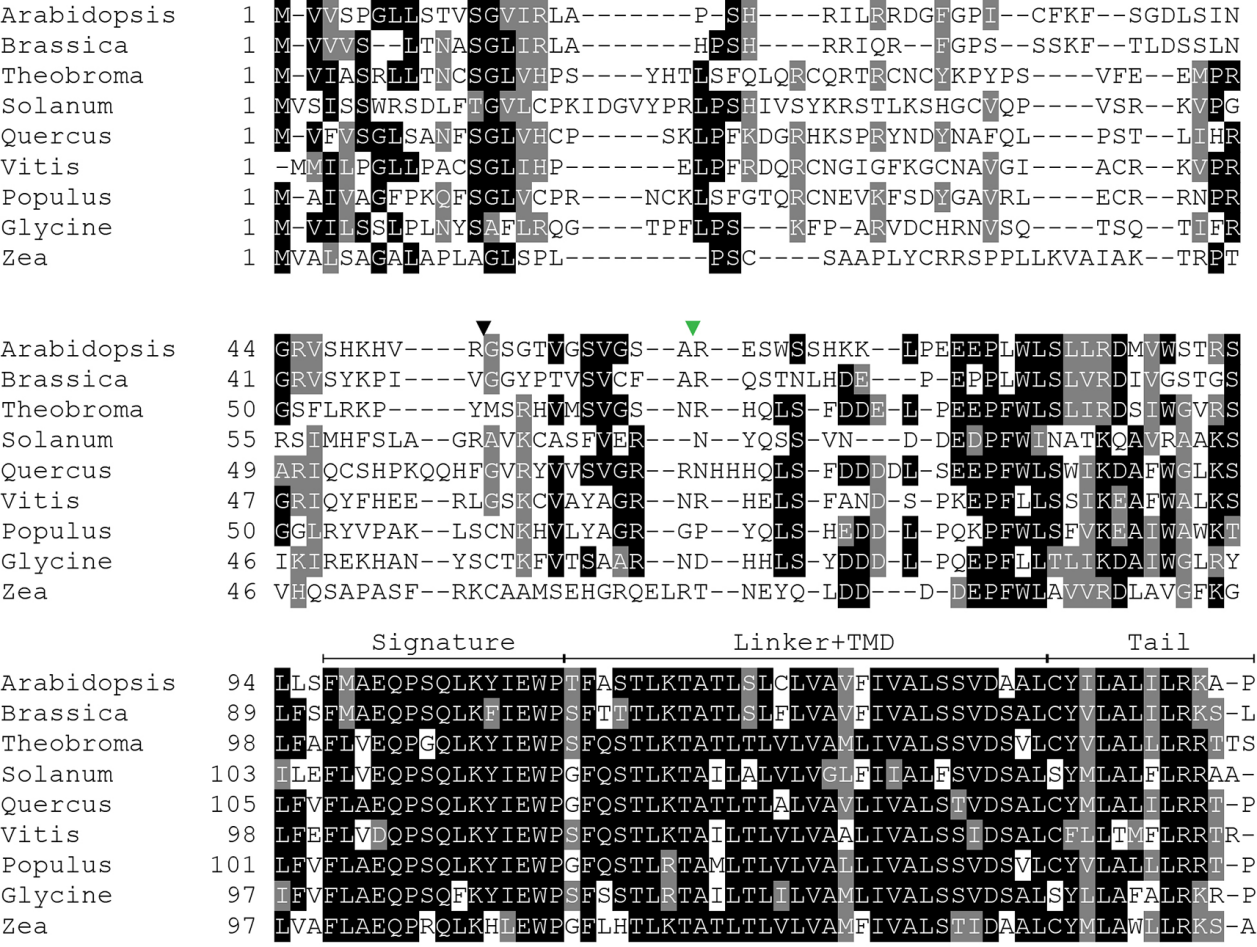
Multiple sequence alignment of SECE2 from a variety of plant species. The predicted transit peptide cleavage site in Arabidopsis is indicated with a black triangle. The GFP insertion site is indicated by a green triangle. Signature, signature region; TMD, transmembrane domain; Tail, C-terminal tail.

Because SECE1 and SECE2 have similar domain structures and topologies, individual domains can be swapped without perturbing the overall structure of the proteins. We undertook a series of domain-swapping experiments in order to identify regions responsible for differential targeting of SECE1 and SECE2. As a first step, we analyzed and aligned SECE1 and SECE2 sequences in different plant species (**Figures 2** and **3**) and identified four regions of interest, which we designated as the N-terminal region (subdivided into N1 and N2), the signature domain, the linker and TMD region, and the C-terminal region (tail). The amino acids included in each region are shown in **Figure 1E**.

The N-terminal region is the region that is least conserved between species, and the SECE1 and SECE2 N-terminal regions cannot be easily aligned. This region is likely to be fully exposed to the stroma following membrane insertion of the TMD. For experimental purposes, we subdivided the N-terminal region into two unequal halves. The region designated as N1 includes the transit peptide and the GFP insertion site. This region lacks any predicted secondary structure according to the JPred4 algorithm (http://www.compbio.dundee.ac.uk/jpred/) (Drozdetskiy et al., 2015). The region designated as N2 includes amino acid sequence predicted to form a helix in both SECE1 and SECE2 (data not shown).

Following the N-terminal region, SECE1 and SECE2 each has a 15-amino acid region designated as the signature domain. When SECE1 and SECE2 are considered separately, the signature domain is highly conserved between different species (**Figures 2** and **3**); however, when SECE1 and SECE2 are compared, their signature domains are quite different. This region ends with the amino acid motif E-W-P, which are the only invariant amino acids in both proteins. A short linker sequence connects the signature region with the predicted TMD. The linker sequences were included with the TMD as a single domain. The TMD is followed by a short region, which we designated as the tail region. We designated the final 18 amino acids as the SECE1 tail and the final 12 amino acids as the SECE2 tail. With three acidic and two basic residues, the SECE1 tail has an overall charge that is slightly negative, while the SECE2 tail has two adjacent basic residues and is positively charged.

### Targeting Determinants Are Associated With the TMDs and C-Terminal Tails

**Figure 4:**
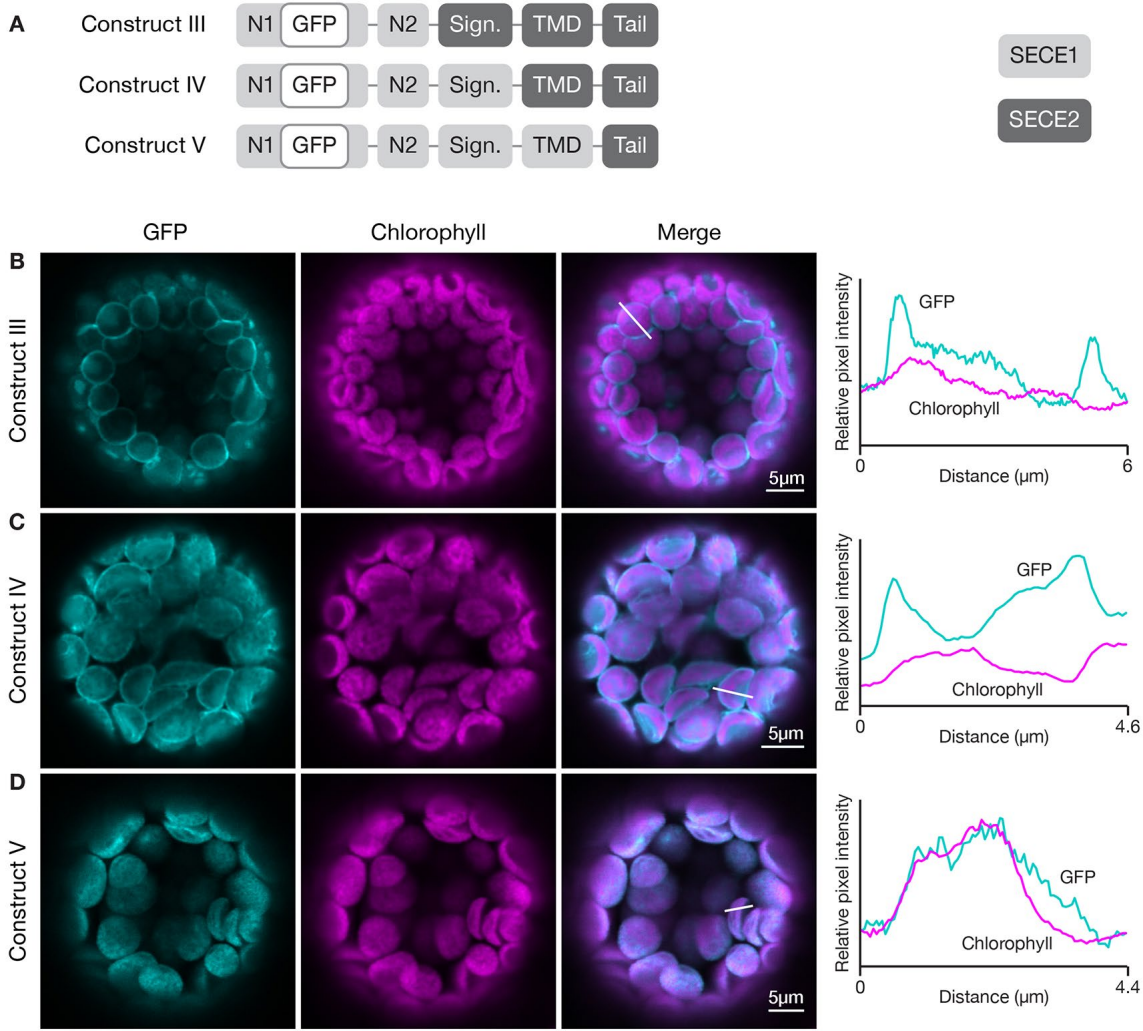
Localization of fluorescent chimeric proteins containing N-terminal SECE1 regions and C-terminal SECE2 regions in transfected protoplasts. **(A)** Diagrams depicting constructs III, IV, and V. SECE1 sequences are indicated with light gray and SECE2 sequences with dark gray; N1 and N2: N-terminal region; GFP: green fluorescent protein; Sign.: signature domain; TMD: linker plus transmembrane domain; Tail: C-terminal tail. **(B**–**D)** Leaf protoplasts from 4–5 week old wildtype (Columbia ecotype) plants were transfected with **(B)** construct III, **(C)** construct IV, or **(D)** construct V. The images show GFP fluorescence (cyan), chlorophyll fluorescence (magenta), merged images, and relative pixel intensity diagrams that correspond with the white lines on the merged images.

To define the minimal region required to target a protein to the inner envelope, we started with full-length GFP-SECE2 and replaced individual regions with the corresponding SECE1 sequence, progressing from the N-terminus to C-terminus of SECE2 (**Figure 4A**). When the N1 and N2 regions of SECE2 were replaced with the corresponding SECE1 sequence, most of the chimeric proteins were still associated with the envelope (construct III, **Figure 4B**). A similar pattern was seen when the N1 region, N2 region, and the signature domain of SECE2 were replaced (construct IV, **Figure 4C**). We concluded that the domains that are predicted to be stroma-exposed after integration were unlikely to contain important determinants for envelope targeting.

When the TMD region of SECE2, in addition to N1 region, N2 region, and the signature domain, was replaced, leaving SECE2 sequence only in the tail (construct V), the chimeric proteins were displaced from the envelope and accumulated in the interior of the chloroplasts (**Figure 4D**). The overlap between GFP fluorescence and chlorophyll autofluorescence, as shown in the fluorescence intensity diagram, suggests that this chimeric protein is associated with the thylakoid membranes. This series of experiments indicated that important inner envelope targeting determinants are associated with the TMD of SECE2.

**Figure 5:**
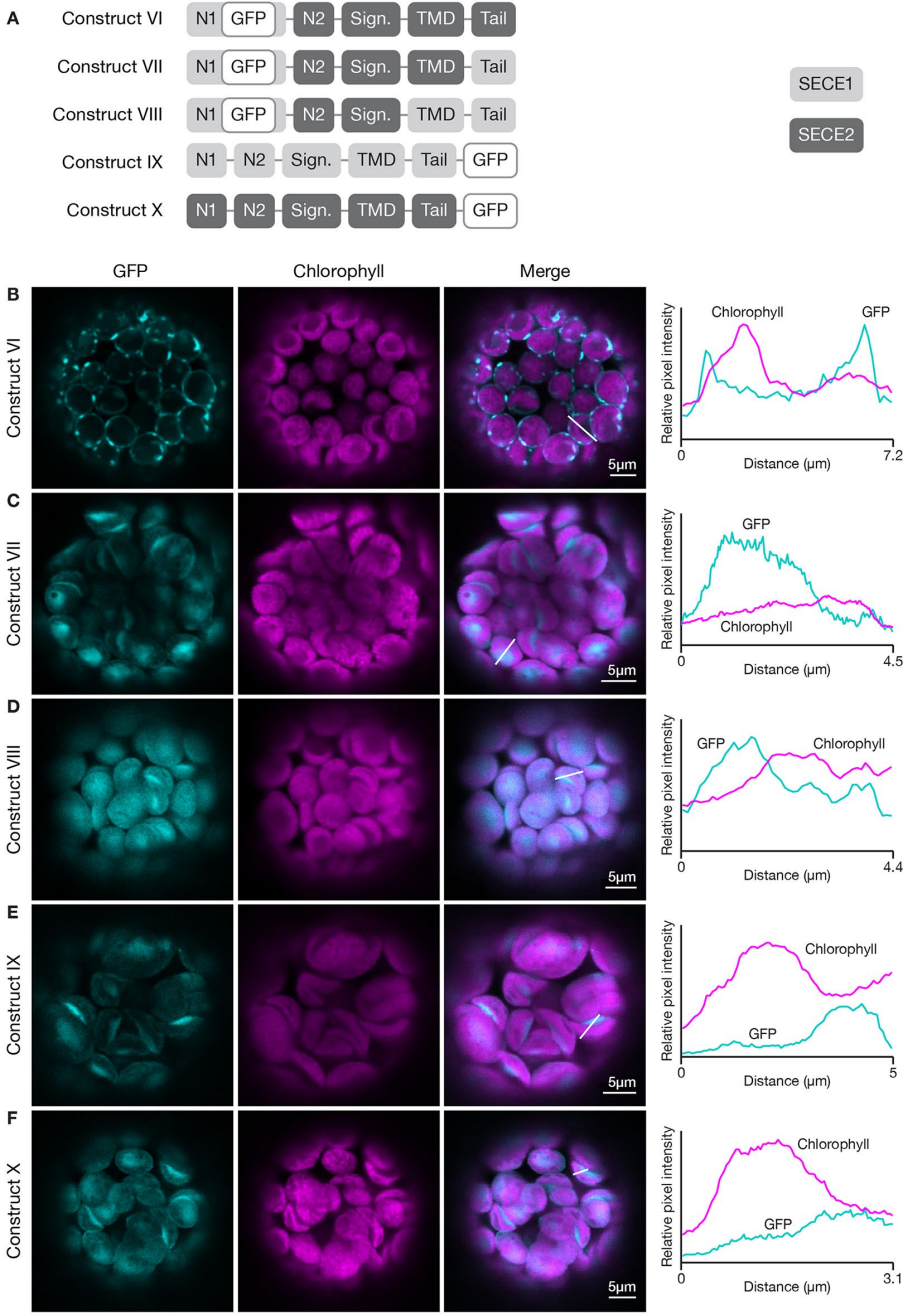
Localization of fluorescent chimeric SECE1 and SECE2 proteins in transfected protoplasts. The SECE1 N1-region is included in constructs VI–VIII to facilitate chloroplast import. **(A)** Diagrams depicting constructs VI, VII, VIII, IX, and X. SECE1 sequences are indicated with light gray and SECE2 sequences with dark gray; N1 and N2: N-terminal region; GFP: green fluorescent protein; Sign.: signature domain; TMD: linker plus transmembrane domain; Tail: C-terminal tail. **(B**–**F)** Leaf protoplasts from 4–5 week old wildtype (Columbia ecotype) plants were transfected with **(B)** construct VI, **(C)** construct VII, **(D)** construct VIII, **(E)** construct IX, or **(F)** construct X. The images show GFP fluorescence (cyan), chlorophyll fluorescence (magenta), merged images, and relative pixel intensity diagrams that correspond with the white lines on the merged images.

To probe this further, we wanted to conduct the inverse experiment, progressively replacing SECE2 sequences with SECE1 sequences starting at the C-terminus. However, we learned in preliminary experiments that chimeric proteins with the N1 region, N2 region, and signature domain of SECE2, and the TMD and tail of SECE1, failed to import into the chloroplast (data not shown). We found that if we modified the constructs such that they included the N1 region of SECE1, the chimeric proteins were successfully imported. Therefore, we started this series of experiments with a construct that encoded most of the mature SECE2 protein but included the N1 region of SECE1 (construct VI, **Figure 5**). The chimeric protein encoded by this construct was primarily associated with the envelope (**Figure 5B**). We observed some fluorescent foci, which we suspect reflects a limited degree of protein aggregation. Despite these visible foci, this protein retained the overall localization pattern of full length SECE2. When we replaced the SECE2 tail with the SECE1 tail (construct VII), the chimeric protein showed stromal accumulation rather than envelope localization (**Figure 5C**). Chimeric proteins that included both the SECE1 TMD and tail (construct VIII) also accumulated primarily in the stroma (**Figure 5D**). We conclude that the SECE2 tail is also necessary for envelope targeting.

Next, we asked whether the sequences in the TMD and tail must be located at the C-terminus in order to function appropriately as targeting determinants. To test this, we fused sequence encoding GFP to the 3’ end of the coding sequence for full-length SECE1 and SECE2, creating SECE1-GFP and SECE2-GFP (constructs IX and X, **Figure 5A**). When SECE1-GFP was expressed in protoplasts, it was efficiently imported and accumulated in the stroma (**Figure 5E**). Similar results were obtained with SECE2-GFP (**Figure 5F**). From these experiments, we concluded that the targeting determinants associated with the TMD and tail are context-dependent, i.e., they must be near the C-terminus to successfully target a TA protein to the appropriate target membrane.

### TMD Regions Alone Are Not Sufficient to Confer Either Envelope or Thylakoid Targeting

Studies of cytosolic TA proteins indicated that physicochemical features of the C-terminal regions, such as TMD hydrophobicity, helical propensity, and charges in the tail region, are important for targeting (Hwang et al., 2004; Kriechbaumer et al., 2009; Rao et al., 2016; Teresinski et al., 2019; reviewed in Chio et al., 2017). If TA protein targeting within chloroplasts depends on the same features, we might expect the TMDs of SECE1 and SECE2 to differ significantly with regard to at least one of these parameters. Because TMD sequence predictions depend on the algorithm used, we analyzed three different predicted TMD sequences for each protein (**Table 1**). These were obtained either using the ConPred_v2 and AramTmCon algorithms [available through the Aramemnon database (http://aramemnon.uni-koeln.de/)] or the topology prediction in the UniProt database (https://www.uniprot.org/). The latter predicts a TMD of 21 amino acids for SECE1 and a longer TMD of 30 amino acids for SECE2. Grand Average of Hydropathy (GRAVY) scores were obtained using the GRAVY calculator (http://www.gravy-calculator.de/). GRAVY scores for the TMD of SECE1 varied from 1.776 to 2.31, while GRAVY scores for the TMD of SECE2 varied from 2.2 to 2.38. Both proteins would be considered to have moderate hydrophobicity when compared to the values obtained with cytosolic TA proteins in yeast, whose GRAVY scores vary from 0.87 to 3.73 (Rao et al., 2016) Therefore, despite possible differences in length, the TMDs of SECE1 and SECE2 do not appear to differ significantly in overall hydrophobicity.

**Table 1:** Grand Average of Hydropathy (GRAVY) and helical propensity (Agadir) scores for predicted transmembrane domains (TMDs) of SECE1 and SECE2.

Prediction method	SECE1 predicted TMD At4g14870/O23342-1	GRAVY score	Agadir score
ConPredv2	GVVLGVIAGSSVVLLTVNFLL	2.205	0.29
AramTmCon	TTGVVLGVIAGSSVVLLTVNF	1.776	0.26
UniProt	VVLGVIAGSSVVLLTVNFLLA	2.31	0.27
**Prediction method**	**SECE2 At4g38490/Q940H5-1**	**GRAVY score**	**Agadir score**
ConPredv2	TLSLCLVAVFIVALSSVDAAL	2.2	2.12
AramTmCon	LCLVAVFIVALSSVDAALCYI	2.362	2.04
UniProt	ATLSLCLVAVFIVALSSVDAALCYILALIL	2.38	6.12

ConPredv2 and AramTmCon predictions were obtained by submitting the gene locus number to the Aramemnon database (http://aramemnon.uni-koeln.de/). The UniProt prediction was obtained by submitting the UniProt ID number to the UniProt database (http://www.uniprot.org). GRAVY scores for each predicted TMD were obtained using the GRAVY calculator (http://www.gravy-calculator.de/), and Agadir scores reflecting helical propensity were obtained using the Agadir algorithm (http://agadir.crg.es/).

Another parameter that is useful for comparing different TMDs is the helical propensity, calculated as Agadir scores (http://agadir.crg.es/about.jsp), which reflect the relative tendency to form helices in aqueous solution. The Adagir score of SECE1’s TMD varies from 0.26 to 0.29, while the Agadir score of SECE2’s TMD varies from 2.04 to 6.12 (**Table 1**). The Agadir scores of cytosolic TA proteins in yeast vary from 0.13 to 74.40. Lower Agadir scores are typically associated with TA proteins targeted to the mitochondrial outer envelope, while higher Agadir scores are typically associated with TA proteins targeted to the ER (Rao et al., 2016). Although the TMD of SECE2 appears to have a slightly higher helical propensity than the TMD of SECE1, the Agadir scores for SECE1 and SECE2 TMDs are both on the low end of the scale.

**Figure 6:**
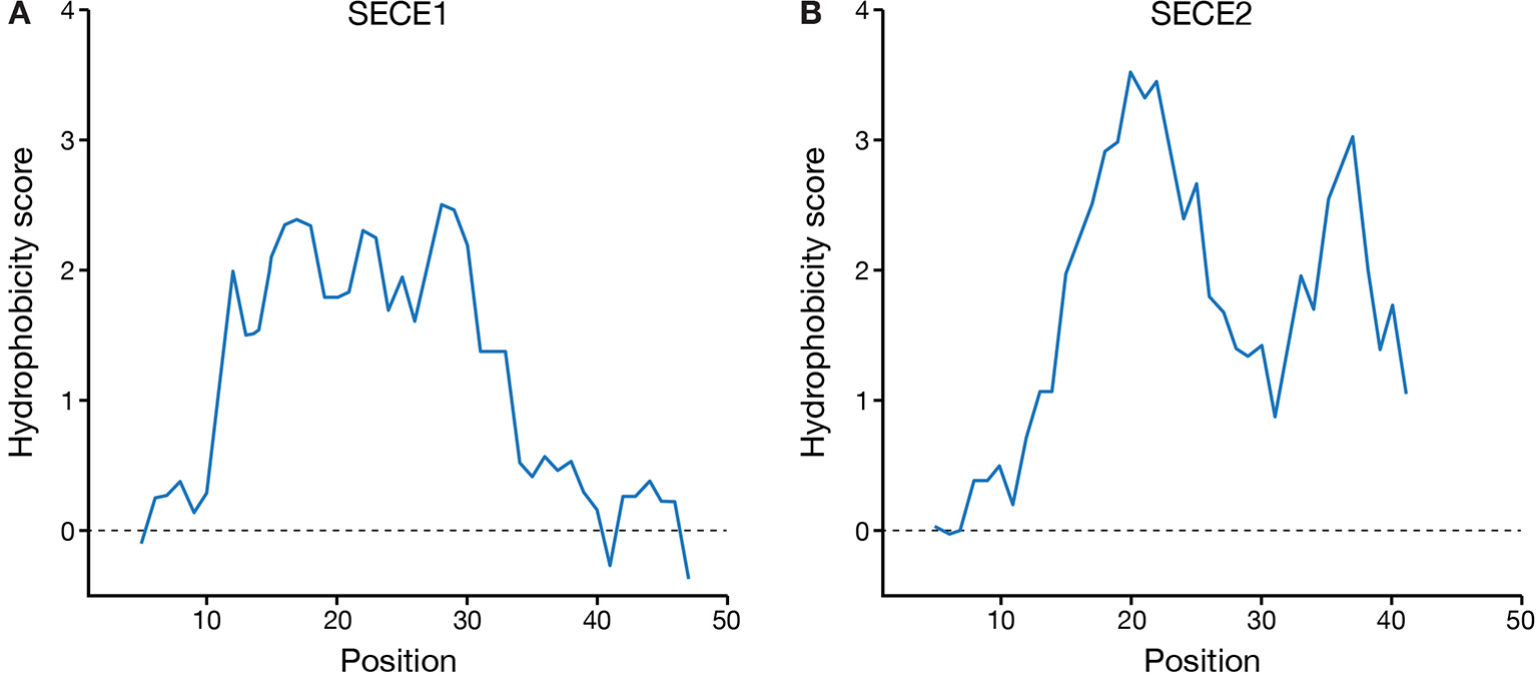
Hydrophobicity profiles of the C-terminal regions of SECE1 and SECE2. Profiles were generated through ExPASy ProtScale (https://web.expasy.org/protscale/) using the Kyte & Doolittle amino acid scale with a window size of 9. Each profile represents the linker, TMD, and tail regions beginning with the E-W-P motif. **(A)** Hydrophobicity profile of SECE1’s C-terminal regions, corresponding to residues 127–177 of the full-length protein. **(B)** Hydrophobicity profile of SECE2’s C-terminal regions, corresponding to residues 109–153 of the full-length protein.

Finally, we compared the hydrophobicity profiles of the TMD and tail regions of SECE1 and SECE2 (**Figure 6**). SECE1’s TMD shows fairly uniform hydrophobicity throughout its length and is followed by a tail with low hydrophobicity. The TMD and tail regions of SECE2, on the other hand, have two “peaks” of higher than average hydrophobicity separated by a “valley” of lower than average hydrophobicity, which is centered around the charged amino acid in the TMD. Therefore, although the GRAVY scores of the TMDs of the two proteins are similar, the hydrophobicity profiles are very different.

**Figure 7:**
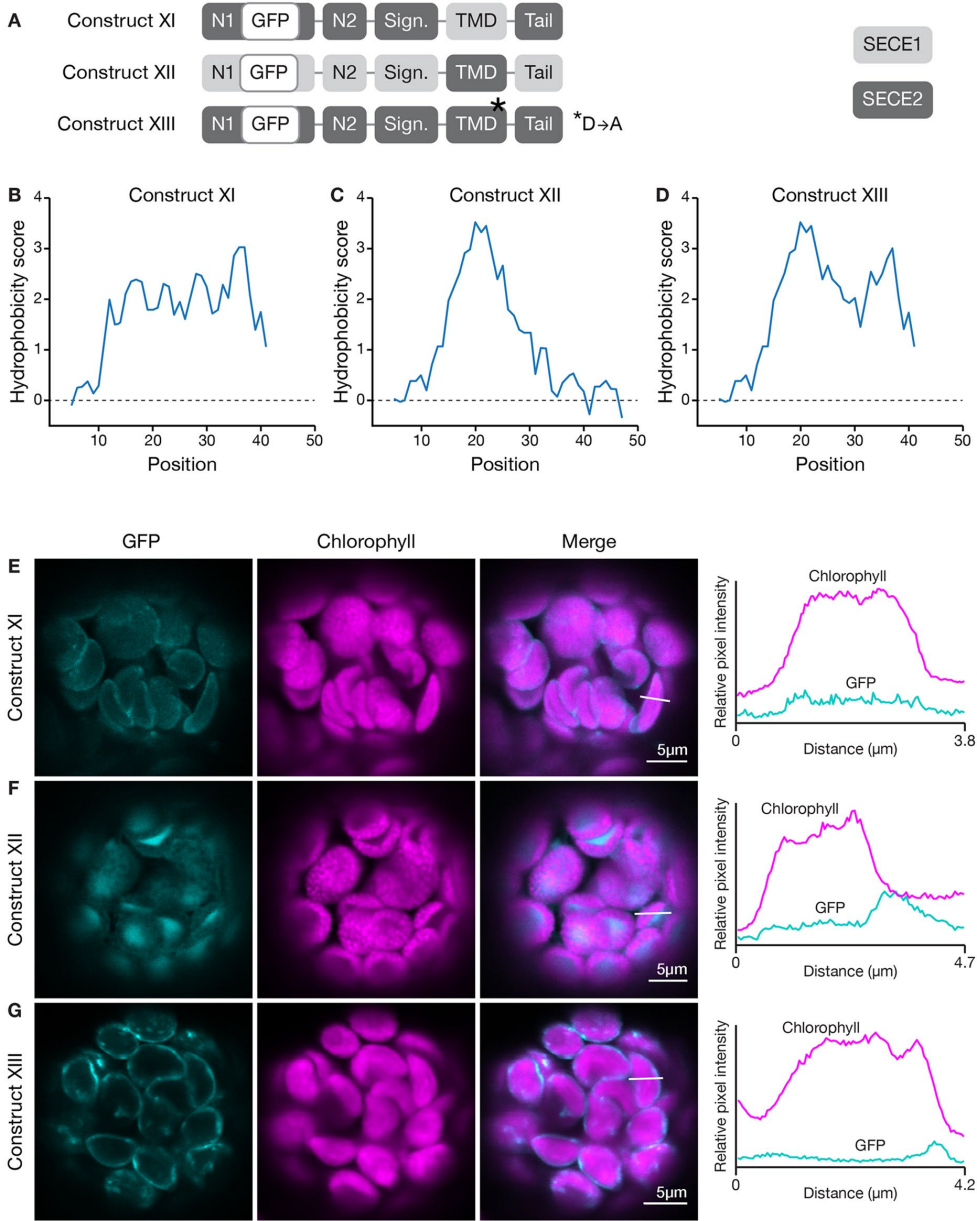
Localization of fluorescent chimeric SECE1 and SECE2 proteins with alterations in the TMD region. **(A)** Diagrams depicting constructs XI, XII, and XIII. SECE1 sequences are indicated with light gray and SECE2 sequences with dark gray; N1 and N2: N-terminal regions; GFP: green fluorescent protein; Sign.: signature domain; TMD: linker plus transmembrane domain; Tail: C-terminal tail. The asterisk indicates the location of a D to A amino acid substitution. **(B**–**D)** Hydrophobicity profiles of the linker, TMD, and tail regions of the proteins encoded by **(B)** construct XI, **(C)** construct XII, and **(D)** construct XIII. **(E–G)** Leaf protoplasts from 4–5 week old wildtype (Columbia ecotype) plants were transfected with **(E)** construct XI, **(F)** construct XII, or **(G)** construct XIII. The images show GFP fluorescence (cyan), chlorophyll fluorescence (magenta), merged images, and relative pixel intensity diagrams that correspond with the white lines on the merged images.

How does the character of the TMD affect targeting? To determine this, we generated a construct encoding a chimeric protein where the TMD of SECE1 was placed in the context of SECE2 (construct XI, **Figure 7A**) and another where the TMD of SECE2 was placed in the context of SECE1 (construct XII, **Figure 7A**). These changes resulted in significant alterations in the hydrophobicity profiles of the TMD and tail regions (compare **Figures 7B, C** to **Figures 6A, B**). The chimeric protein encoded by construct XI had uniform moderate hydrophobicity throughout the TMD and tail regions (**Figure 7B**). When protoplasts were transfected with this construct, only weak fluorescence was seen (**Figure 7E**). The chimeric protein did not appear to show any specificity and low-level fluorescence was associated with multiple locations, including the thylakoid, stroma, and envelope. For the chimeric protein encoded by construct XII, the first peak of higher hydrophobicity associated with SECE2’s TMD was retained but the second peak was eliminated (**Figure 7C**). The chimeric protein produced by this construct primarily accumulated in the stroma (**Figure 7F**). We conclude that the TMD regions alone are not sufficient to confer either envelope or thylakoid targeting, possibly because physiochemical features of the C-terminal region are shaped by contributions from both the TMDs and tail regions.

The valley between the two peaks in the hydrophobicity profile of SECE2 is centered on a charged residue (aspartic acid). We tested whether changing this amino acid has an impact on targeting. We generated a GFP-SECE2 construct in which the aspartic acid was replaced by an alanine residue (construct XIII, **Figure 7A**). This amino acid substitution results in minimal change to the hydrophobicity profile. The floor of the valley between the two high peaks of hydrophobicity associated with SECE2 is raised but the valley is not eliminated (compare **Figure 7D** to **Figure 6B**). When protoplasts were transfected with this construct, the fluorescent mutant protein was targeted primarily to the envelope (**Figure 7G**). Thus, having a charged residue in this position may be less important for envelope targeting than the overall hydrophobicity profile.

### C-Terminal Tail Characteristics Play a Role in Membrane-Specific Targeting

**Figure 8:**
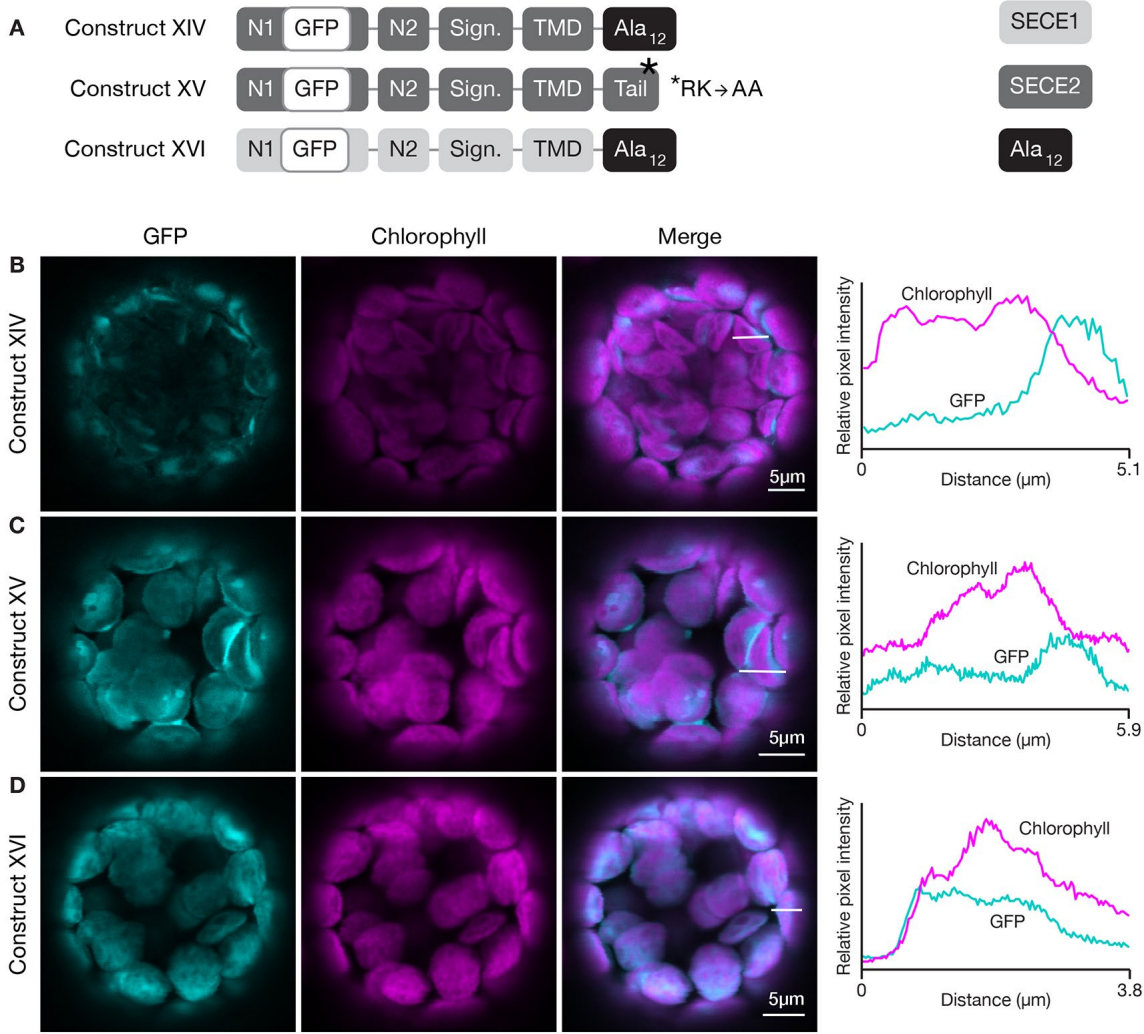
Localization of fluorescent chimeric SECE1 and SECE2 proteins with alterations in the C-terminal tail regions. **(A)** Diagrams depicting constructs XIV, XV, and XVI. SECE1 sequences are indicated with light gray and SECE2 sequences with dark gray; N1 and N2: N-terminal regions; GFP: green fluorescent protein; Sign.: signature domain; TMD: linker plus transmembrane domain; Tail: C-terminal tail. The black rectangles with Ala_12_ indicate a substitution of the entire native tail with 12 alanine residues. The asterisk indicates the location of an R-K to A-A amino acid substitution. **(B–D)** Leaf protoplasts from 4–5 week old wildtype (Columbia ecotype) plants were transfected with **(B)** construct XIV, **(C)** construct XV, or **(D)** construct XVI. The images show GFP fluorescence (cyan), chlorophyll fluorescence (magenta), merged images, and relative pixel intensity diagrams that correspond with the white lines on the merged images.

The results of the first two sets of domain swap experiments suggested that the character of the tail region was more important for envelope-directed targeting than for thylakoid-directed targeting. Substitution of SECE1’s tail for the tail of SECE2 blocked association of SECE2 with the envelope membrane, while substitution of SECE2’s tail for the tail of SECE1 was tolerated. The net charge of the tail has been shown to be an important factor for sorting cytosolic tail-anchored proteins (reviewed in Moog, 2019 and Pedrazzini, 2009). The SECE1 tail has three acidic and two basic residues, while the SECE2 tail has two adjacent basic residues. All of the charged residues are highly conserved. We performed several different experiments to test whether changes in the amino acid composition of the SECE2 tail would affect localization to the envelope. First, we generated a construct encoding a SECE2 protein where the final 12 amino acids were changed to alanine residues (construct XIV, **Figure 8A**). When protoplasts were transfected with this construct, the fluorescent mutant protein accumulated in the stroma (**Figure 8B**). Next, we tested a construct where the dibasic R-K motif was changed to A-A (construct XV, **Figure 8A**). This fluorescent mutant protein also accumulated in the stroma (**Figure 8C**). Similar results were obtained with constructs encoding proteins where either the first six residues in the tail were changed to alanines or the last six residues (including the R-K motif) were changed to alanines (data not shown). We conclude that there are stringent requirements for particular tail sequences for efficient envelope-directed SECE2 targeting.

We also tested the effect of alterations in the amino acid composition of the tail on thylakoid-directed targeting. A construct was generated that encoded a SECE1 protein where the entire tail was replaced by 12 alanine residues (construct XVI, **Figure 8A**). In transfected protoplasts, the fluorescent mutant protein was associated with the thylakoids (**Figure 8D**). Therefore, the native tail, including its two positive and three negative charged residues, is clearly not required in the context of full-length SECE1. In fact, in contrast to envelope-directed SECE2 targeting, we could not detect any requirements for particular tail sequences for thylakoid-directed SECE1 targeting.

In summary, these experiments show that, just as in the cytosolic targeting systems for TA proteins, targeting of TA proteins to internal chloroplast membranes depends on determinants associated with the TMD and tail regions. Targeting to the thylakoids and targeting to the inner envelope have somewhat different requirements. For envelope targeting, the TMD and tail regions are both important, and adjacent basic residues or some other feature of the tail may be necessary for high fidelity targeting. For thylakoid targeting, the TMD and tail regions are also important; however, in the context of full-length SECE1, the requirement for the native tail is relaxed.

## Discussion

### Summary Model

**Figure 9:**
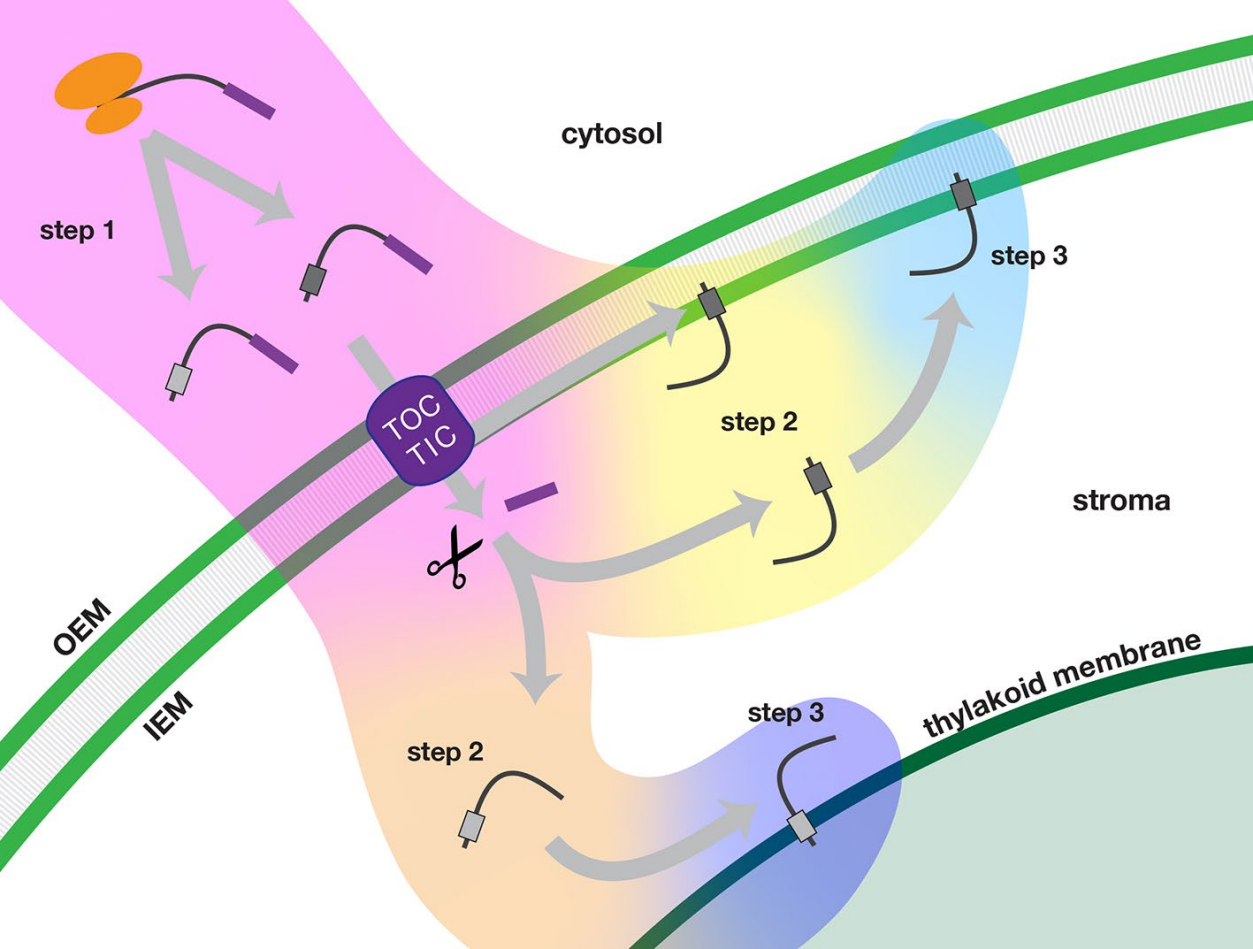
Multi-step model of targeting nuclear-encoded TA proteins to the inner envelope or thylakoid membranes of chloroplasts. In step 1 (pink background color), the TA protein is synthesized in the cytosol and delivered to the chloroplast. In step 2, the TA proteins are sorted either in an inner envelope-directed (yellow background color) or thylakoid-directed (peach background color) pathway. In step 3, the TA proteins are integrated either into the inner envelope membrane (light blue background color) or the thylakoid membrane (dark blue background). Scissors indicate stromal processing peptidase activity. IEM, inner envelope membrane; OEM, outer envelope membrane; TOC/TIC, Translocons of the Outer and Inner Envelope membranes of the chloroplast.

TA proteins that are imported and localized in either the inner envelope or thylakoid membranes of chloroplasts require a number of targeting signals for accurate delivery and successful integration. Our studies show that membrane specificity depends primarily on signals located at the C-terminus, in the TMD and tail regions. In **Figure 9**, we present a multistep model for how the nuclear-encoded imported chloroplast TA proteins are targeted.

In step 1, precursor TA proteins are synthesized in the cytosol with transit peptides. The newly synthesized precursors evade the cytosolic TA protein targeting systems and are delivered to the TOC-TIC import apparatus of the chloroplast. Following import of the N-terminus, the transit peptide of the precursor protein is removed in the stroma.

In step 2, TA proteins are recruited into an inner envelope-directed pathway or a thylakoid-directed pathway, based on characteristics of their TMD and tail regions. Inner envelope TA proteins have two possible routes: translocation may be arrested during import and the protein may be released laterally from the TIC. Alternatively, they may take a post-import route, as do thylakoid TA proteins. According to our model, fully imported TA proteins would be recruited to the appropriate pathway through interactions with stromal factors as the TMD and tail regions exit the TIC.

In step 3, the TA proteins are integrated into the target membranes. Insertion may be spontaneous or it may be catalyzed by a specific translocase. The efficiency of integration may also depend on the characteristics of the TMD and tail regions.

### Pre- and Post-Import Targeting Features Are Located on Distinct Termini

Targeting of internal chloroplast TA proteins involves at least two sets of targeting determinants: one set at the N-terminus for delivery to the chloroplast import machinery and one set close to the C-terminus for localization within the chloroplast. The N-terminal determinants appear to be dominant in the cytosol, perhaps because this region is exposed first during synthesis, while the C-terminal regions are still in the ribosome tunnel. Interactions between cytosolic factors and transit peptide sequences that result in the precursor proteins being directed to the TOC-TIC import apparatus have been extensively studied (May and Soll, 2000; Qbadou et al., 2006; Bölter and Soll, 2016).

Following import and removal of the transit peptide, determinants near the C-terminus become important, just as they are in the cytosolic TA protein targeting systems. Both SECE1 and SECE2 have TMDs with moderate hydrophobicity, but the tails of SECE1 and SECE2 are quite different. Both tails lack the RK/ST-enriched sequences associated with the tails of many chloroplast outer envelope TA proteins (Teresinski et al., 2019). In the case of SECE2, the tail region most closely resembles the targeting sequence of a mitochondrial outer envelope TA protein. Mitochondrial TA proteins tend to have tails with a more positive net charge and often contain a dibasic R-R/K/H-X (X≠E) motif (Hwang et al., 2004; Marty et al., 2014). In Arabidopsis and other species, the sequence R-R/K-X, where X tends to be either A, T, or S, is highly conserved in SECE2’s tail (**Figure 3**). We showed that changing the R-K motif to A-A disrupts targeting of SECE2 to the inner envelope. In the case of SECE1, the tail region most closely resembles the targeting sequence of ER-directed TA proteins, which often contain a R/H-X-Y/F motif and have a slightly positive net charge (Moog, 2019). SECE1’s tail has a slightly negative net charge instead, but it contains the highly conserved R-V-F motif in all species we examined aside from *Zea*, where the sequence is T-V-F (**Figure 2**). Because replacing the tail with A residues or substituting SECE2’s tail for the SECE1 tail does not block the thylakoid localization of SECE1, these residues may be dispensable for targeting.

We are not suggesting that the inner envelope is equivalent to the mitochondrial outer envelope, nor are the thylakoids equivalent to the ER. However, the parallelism is curious and raises the possibility that physicochemical features that contribute to discrimination between different pathways in the cytosol might serve a similar purpose in the stroma.

### Post-Import Sorting Events

Following import into the stroma and removal of the transit peptide, the TA proteins are sorted into different targeting pathways. For thylakoid-directed proteins, interaction with one or more stromal factors is likely necessary to prevent aggregation and aid in delivery to the thylakoids. Based on the observation that SECE1 can integrate into isolated thylakoids in the absence of stromal extracts and an energy source (Steiner et al., 2002), SECE1 is often considered to be a client of the “spontaneous” pathway. However, given the similar lipid composition of the thylakoids and inner envelope (Benning, 2009), it is hard to reconcile a protein-free targeting process with the membrane specificity that is observed *in vivo*. We suggest that targeting to the thylakoid is more likely to be protein-mediated. Based on its sequence, SECE1 does not appear to be a good candidate for either the SEC, ΔpH, or SRP pathways. It lacks the N-terminal sequences that resemble signal peptides often found in clients of the SEC and ΔpH pathways, and it lacks the D-P-L-G motif used to recruit proteins to the SRP pathway (DeLille et al., 2000). Given the resemblance of SECE1’s tail to those of ER-directed TA proteins, which are delivered *via* the cytosolic Guided Entry of Tail-anchored proteins (GET) system, we speculate that targeting of thylakoid TA proteins might be a possible role for GET3B, the recently discovered chloroplast homolog of the GET3 targeting factor (Duncan et al., 2013; Xing et al., 2017).

For inner envelope TA proteins, there are two possible targeting pathways. The final topology, with a stroma-exposed N-terminus, would be compatible with a stop transfer mechanism, which involves arrest of translocation and lateral release of the TMD from the TIC. Features previously identified as being associated with stop transfer TMDs include absence of proline residues, a relatively high content of clustered tryptophan and phenylalanine residues, and G-X_3_-G or G-X_4_-G motifs (Froehlich and Keegstra, 2011). SECE2’s TMD lacks proline residues, but it does not have any other feature that would suggest it functions as a stop transfer TMD. Our experiments have shown that SECE2’s TMD must be combined with the appropriate tail to be an effective targeting determinant (compare constructs VII, XII, and XIV to construct II). Therefore, it is unlike the single-span inner envelope protein ARC6, whose stop transfer TMD is sufficient to confer inner envelope localization (Froehlich and Keegstra, 2011). We found that full-length SECE2 proteins with GFP fused at their C-terminus and several different mutant SECE2 proteins with amino acid changes in the tail region accumulate in the stroma (constructs X, XIV, XV). It is possible that the amino acid changes interfere with the arrest of translocation or some other aspect of the stop transfer mechanism. However, it is equally possible that SECE2 normally fully transits the TIC, and the C-terminal extensions and/or alterations alter the dynamics of the normally transient stromal pool, perhaps by slowing or preventing integration from the stromal side of the inner envelope.

If both SECE2 and SECE1 use post-import pathways, what features of the TMDs and tails might contribute to sorting? As discussed in the previous section, the tails of SECE1 and SECE2 carry different net charges. The tail appears to be more important for inner envelope targeting than for thylakoid targeting. The TMDs of SECE1 and SECE2 are similar in that they have moderate hydrophobicity; however, they differ in several regards: First, SECE1’s TMD has a lower helical propensity than SECE2’s TMD. Helical propensity differences were previously shown to be strongly correlated with the efficacy of targeting of TA proteins to different target membranes in the cytosol (Rao et al., 2016). Second, SECE1’s TMD, but not SECE2’s TMD, has features that are often associated with the TMDs of thylakoid proteins, namely clustered leucine residues and a relatively high alanine (A), valine (V), and glycine (G) content (Froehlich and Keegstra, 2011). Finally, we note that while the average hydrophobicities of SECE1’s and SECE2’s TMDs are similar, the hydrophobicity profiles are very different: SECE1’s TMD shows fairly uniform hydrophobicity throughout its length, and is followed by a tail with multiple charged residues and low hydrophobicity. The TMD and tail regions of SECE2, on the other hand, are marked by two regions of higher hydrophobicity separated by a region of lower hydrophobicity that includes a charged amino acid. Changing the aspartic acid to alanine had no apparent effect on targeting (construct XIII); however, the change also did not completely eliminate the region of lower hydrophobicity in the hydrophobicity profile (compare **Figure 7D** to **Figure 6B**). The difference in SECE1 and SECE2’s profiles could be significant if stromal factors discriminate between clients on this basis. For example, a stromal factor with a hydrophobic groove that accommodates SECE1’s TMD might be unable to accommodate SECE2’s TMD because of the region of lower hydrophobicity or the nearby charges in the tail. Additional experiments will be needed to test which of the many differences between SECE1 and SECE2’s TMD and tail regions are significant.

### Membrane Integration

After successful recruitment into the appropriate pathway and delivery to the target membrane, the TA protein must be integrated. Integration involves partitioning of the hydrophobic TMD into the lipid bilayer and transfer of the tail region across the lipid bilayer. The thylakoids contain a variety of different translocases, any of which could at least theoretically be involved in this process. Because thylakoids are an energized membrane, transfer also might be aided by the proton-motive force. Therefore, thylakoids clearly have the capacity to transfer a small protein domain such as a short C-terminal tail across the membrane. Indeed, it was reported previously that SECE1 can insert into isolated thylakoid membranes without stromal extracts or an energy source (Steiner et al., 2002) and we observed that successful integration can occur with a variety of different short tails (construct V and XVI). For inner envelope proteins, transfer of the tail across a membrane only becomes a concern for TA proteins that integrate by a post-import mechanism. In this case, we imagine that TA proteins are brought in close proximity to the membrane by targeting complexes, allowing integration to occur. This might happen spontaneously, *via* the SEC2 translocase, or *via* some as-yet-unidentified translocase. The lack of a protonmotive force and more limited capacity for transfer might explain why we see a more stringent requirement for a particular tail with SECE2.

In summary, we have shown that membrane-specific targeting of TA proteins within chloroplasts depends on features of their TMDs and short C-terminal tails. We propose that physicochemical features of the TMDs and tail regions are important in two ways. First, they dictate whether a given tail-anchored protein is recruited to either a thylakoid- or an inner envelope-targeting pathway. Second, after delivery to the target membrane, attributes of the TMDs and tail regions may influence membrane insertion.

## Data Availability Statement

All datasets generated for this study are included in the article.

## Author Contributions

SA, RS, and DF designed and performed the research. SA and DF wrote the manuscript. All authors contributed to manuscript revision, and read and approved the submitted version.

## Funding

Support for this research was provided by the United States National Science Foundation (DGE1747503 to SA, MCB1158173 to DF) and the University of Wisconsin-Madison Office of the Vice Chancellor for Research and Graduate Education with funding from the Wisconsin Alumni Research Foundation.

## Conflict of Interest

The authors declare that the research was conducted in the absence of any commercial or financial relationships that could be construed as a potential conflict of interest.

## Abbreviations

GFP, green fluorescent protein; GRAVY, grand average of hydropathy; TA, tail-anchored; TMD, transmembrane domain
