# Supplementary material for: Membrane-Specific Targeting of Tail-Anchored Proteins SECE1 and SECE2 Within Chloroplasts
Source: Front Plant Sci. 2019 Nov 8;10:1401. doi: 10.3389/fpls.2019.01401 (PMC6857650; doi:10.3389/fpls.2019.01401)
Supplement: Supplementary file 1 [file DataSheet_1.pdf]

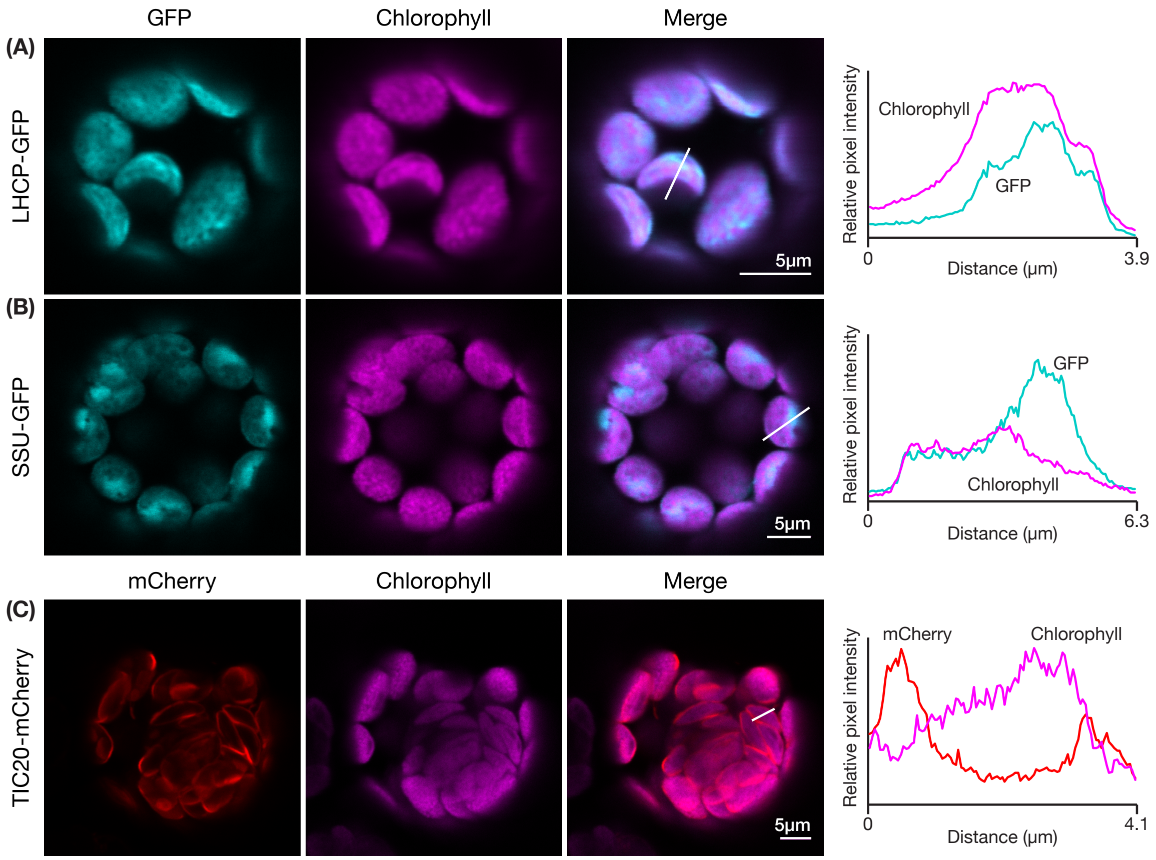

Figure S1. Localization of fluorescent proteins in transfected protoplasts. Leaf protoplasts from 4-5 week old wildtype (Columbia ecotype) plants were transfected with constructs encoding (A) Light harvesting chlorophyll a/b-binding protein (LHCP)-GFP, a marker for thylakoid localization, (B) Small subunit of Rubisco (SSU)-GFP, a marker for stroma localization, or (C) TIC20-mCherry, a marker for inner envelope localization. The images show GFP fluorescence (cyan), mCherry fluorescence (red), or chlorophyll autofluorescence (magenta), merged images, and relative pixel intensity diagrams that correspond with the white lines on the merged images. SSU-GFP and TIC20-mCherry plasmids were obtained from Dr. Enrico Schleiff. The LHCP sequence was amplified from seedling cDNA and corresponds to the gene At1G29920.
