# Supplementary material for: Membrane-Specific Targeting of Tail-Anchored Proteins SECE1 and SECE2 Within Chloroplasts
Source: Front Plant Sci. 2019 Nov 8;10:1401. doi: 10.3389/fpls.2019.01401 (PMC6857650; doi:10.3389/fpls.2019.01401)
Supplement: Supplementary file 2 [file DataSheet_2.pdf]

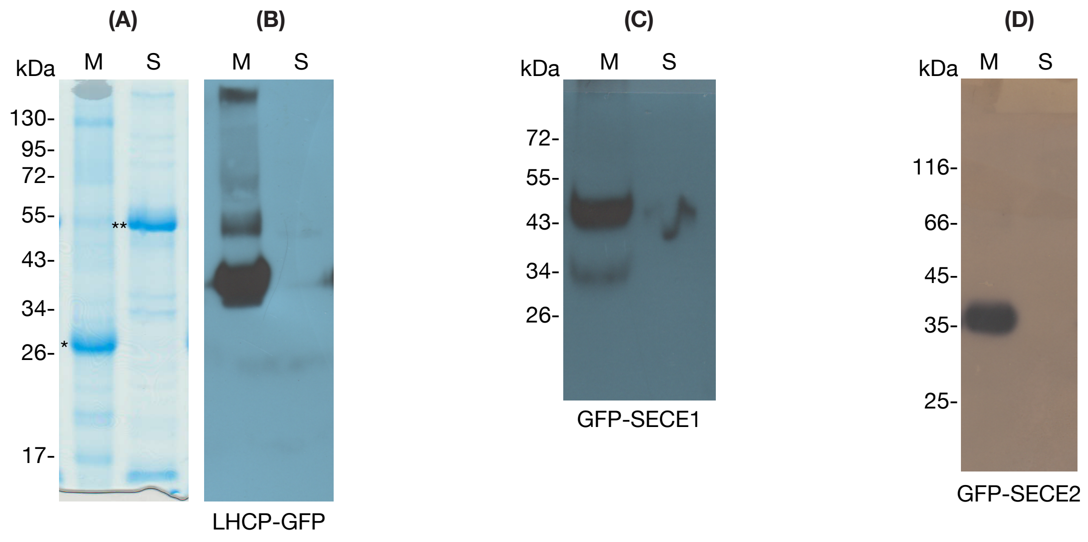

Figure S2. Localization of GFP fusion proteins by immunoblot analysis following fractionation of lysed chloroplasts isolated from leaf protoplasts transfected with constructs encoding various GFP fusion proteins. Chloroplasts were lysed and membrane and soluble fractions were prepared as described in Li et al., Plant J. (2015) 84: 647, except that the membrane fraction was washed with 0.2M Na_2CO_3 before analysis. (A) Coomassie-stained gel showing the protein profiles of membrane (M) and soluble (S) fractions. Major bands corresponding to light harvesting chlorophyll a/b binding proteins (LHCP) (*) and the large subunit of Rubisco (**) can be detected only in the membrane and soluble fractions, respectively. (B-D) Immunoblots probed with GFP antibodies. Fractions were isolated from protoplasts transformed with (B) LHCP-GFP, (C) GFP-SECE1, or (D) GFP-SECE2. LHCP-GFP and GFP-SECE2 are associated with the membrane fraction. GFP-SECE1 is associated primarily with the membrane fraction, although some can be detected in the soluble fraction as well. kDa= kilodaltons.
